# Launched

**Published:** 1885-07

**Authors:** 


### DANIEL'S MEDICAL ★ JOURNAL,

AUSTIN, TEXAS.

Subscription Price: Two Dollars per Annum in Advance.

Papers for publication in this Journal should be in the hands of the Editor by the 10th of the month preceding the month in which it is wished to appear; they must be original,--never having appeared in print elsewhere, and must be contributed exclusively to this Journal.

Contributions to the department of Original Articles are cordially invited from the profession of Texas, especially. What is most desired is Clinical reports of cases, essays and short practical articles on any medical topic ; also solicited reports of proceedings of societies, clubs, etc., and items of medical news of general interest to the profession, such as notices of appointments of physicians, removals, changes, marriages, deaths, etc.

### EDITORIAL.

### LAUNCHED.

There is a sea, deep and dark, whereon sail many craft; a treacherous sea, beneath whose deceptive waters lie buried hopes and fortunes. A sea, smooth to the eye, and inviting; full of promise, and apparently offering pleasant sailing, and smooth passage, yet under whose surface roll currents of ill will, envy and jealousy. Shoals, there are, of indiscretion and bad judgment; while boldly stands out the magnetic rock of Hard Times. On this, many go to pieces, the venturesome and the unseaworthy.

There are, plying the waters of this sea, vessels of many kinds. There are those of heavy tonnage, which make weekly trips, and safe landings; bringing rich cargoes, the products of many minds, and many lands. These are guided by the practiced eye and steady hand of the master navigator. There are very Esmeraldas, of the John Roach build, so to speak, heavy freighted and well ballasted, whose pilots have buffeted many storms, and have learned by experience to steer around these shoals and rocks, on which so many are dashed, early in life; and on which others founder, even after many successful trips. There are, too, lighter craft, which ply in monthly rounds, and whose cargoes are of lighter stuffs, and less precious. There are Brigs, and Barks, and Brig-rigged Schooners, and Schooners, which scud under bare poles, ballastless, and driven by divers breezes. These to the bottom quickly go, and to-day the number that float on the surface are as naught to the many that rest in oblivion, stranded on the beach of incompetency, or rotting in the mud of failure, at the bottom. We, too, are tempted to embark on these waters. 'Tis an inviting sea, yet well do we know its dangers. A brief experience, in which we had smooth sailing, a few voyages, attended with naught but sunshine, blue skies and soft zephyrs, has sharpened our appetite, and we long for a return to those pleasant scenes. Ah! There *is* "a facination which gathers about the beginning of things," as Dr. Merriman says in *Gaillard's Journal*. In this instance it *may* be the facination which lures the silly moth to the candle flame, and we may get our wings singed. We hope not. Yes, like Oliver Twist, we want "more." We are venturesome; we have built us a boat, and, like Robinson Crusoe, have "set it afloat." (Some one may yet exclaim, "Oh! What made you *do* so?")

Like the Egyptian maiden, whose taper-lighted lotus leaf she has just entrusted, with beating heart, to the treacherous tide of the Nile, we to-day watch this tender, slender venture, lighted with *our hopes*, as she glides out on the deceptive waters of this journalistic sea, this dangerous sea. (We were tempted, for the sake of jingling alliteration, to say, "the treacherous tide of the Tiber," but, seeing that most of the Egyptian maidens have removed from the malarial borders of the classic stream, wherein the festive Cassius and Cæsar were wont to plunge in youthful sport, and have taken board with the Prophet up on the more salubrious banks of the Nile; and seeing, also, that lotuses don't grow in the section watered by the Tiber, we had to sacrifice the poetic alliteration to cold geographical facts.)

Yes, our heart beats high with hope! We tremble with fear! Eagerly we open each letter now, with trembling hand, and as, with rapid glance we "catch on" to its contents, we note how the news of the launching of the NEW TEXAS is received by those to whom she will go, timidly tendering her first cargo of miscellaneous freight; those who will watch its course, some, like ourselves, with hope and interest; some with jealous eyes; but most, we fear, with cold indifference; and, as we break each seal, we are greeted with the cold water of discouragement, or the cordial, warm hand-shake of approbation; while our feelings rapidly run the gamut, sounding the bass note of despondency now, now bounding to the shrillest key in the octave of expectancy. Oh, how the hot blood goes coursing, making our temples throb, our heart go "thump," and our head swim with a glad dizziness, as we read this one: "Yes sir, count me in. I wish you every luck and unbounded success that energy and untiring effort can yield, and I pledge you my hearty support and co-operation. Enclosed, find the first year's subscription. I will get you up a club. Meantime call on me if I can serve you, and believe me sincerely yours."

Here now by contrast; here's where the bass note comes in: "Go slow. I am sorry to hear you have drawn out of the C. R., and think you have made a mistake. There is that undefinable something about the "good will" of an established journal that makes me fear you will not succeed in a new undertaking. Times are so hard, and money so scarce, and—and—and—" (No subscription in this one.)

But, how, like a benediction, comes this blessed language, from one whose opinion we regard, and whose friendship we hold dear:

"My dear Doctor Daniel: Do not be discouraged if your best efforts are misconstrued; if your most ardent supporters fail you when you most need them; if evil doers are prosperous, as they generally are, and your efforts seem fruitless and useless; do not give up the fight, but remember that the right must succeed at last, and success will be yours in reality, if you persevere, even though disappointments attend your best endeavors. I know you will have many hours of despondency, unless you realize, at the outset, the difficulties which lie in the path of the journalist, who makes *principle* his guide, and leaves policy to those weak hearts who have not the faith to see that the good can be reached only through dangers and difficulties. You have my warmest wishes for your success."

There! that is inspired! and coming so opportunely, it has all the soothing and sustaining influence of heavenly prayer for one's safe and prosperous voyage; or like a blessing; or "like the benediction that follows after prayer." Sustained and encouraged by *such* words of cheer, and they are pouring in by every mail, accompanied too, in many cases, by more substantial evidences of good will, we have made all shipshape with the printers, clued down the hatches by advance payments, sounded our fog-horn of a prospectus to let 'em know we are coming, spread our sails, cut loose the guy ropes and stays of indecision, and she glides off the docks with a splurge and a splash! A bottle of the wine of "good hope" has been broken on her prow; the horse-shoe of "good luck" has been thrown at her, and the NEW TEXAS MEDICAL JOURNAL is afloat. Adieu! Adieu, frail boat! May favoring winds waft thee; may smooth waters bear thee; may glad smiles greet thee, and friendly hands speed thee on thy way! And, each trip, may you add to the troop of those who watch thee with friendly eyes, and are glad when thy white sail and the Texas Star heave in sight!

